# Cryochemically Processed Li_1+y_Mn_1.95_Ni_0.025_Co_0.025_O_4_ (y = 0, 0.1) Cathode Materials for Li-Ion Batteries

**DOI:** 10.3390/ma11071162

**Published:** 2018-07-08

**Authors:** Ofok O. Normakhmedov, Oleg A. Brylev, Dmitrii I. Petukhov, Konstantin A. Kurilenko, Tatiana L. Kulova, Elena K. Tuseeva, Alexander M. Skundin

**Affiliations:** 1 Department of Materials Science, Lomonosov Moscow State University, Leninskie Gory, 1-73, Moscow 119991, Russia; normahmedov57@gmail.com (O.O.N.); brylev@inorg.chem.msu.ru (O.A.B.); 2 S.U. Umarov Physical and Technical Institute, Academy of Sciences of Republic of Tajikistan, Aini street 299, Dushanbe 734063, Tajikistan; 3 Department of Chemistry, Lomonosov Moscow State University, Leninskie Gory, 1-3, Moscow 119991, Russia; kostik.msu@mail.ru; 4 A.N. Frumkin Institute of Physical Chemistry and Electrochemistry, Russian Academy of Sciences, 31-4 Leninskii Prospect, Moscow 119071, Russia; tkulova@mail.ru (T.L.K.); elenatusseeva@mail.ru (E.K.T.); askundin@mail.ru (A.M.S.)

**Keywords:** lithium-ion batteries, cathode materials, freeze-drying, LiMn_2_O_4_

## Abstract

A new route for the preparation of nickel and cobalt substituted spinel cathode materials (LiMn_1.95_Co_0.025_Ni_0.025_O_4_ and Li_1.1_Mn_1.95_Co_0.025_Ni_0.025_O_4_) by freeze-drying of acetate precursors followed by heat treatment was suggested in the present work. The experimental conditions for the preparation single-phase material with small particle size were optimized. Single-phase spinel was formed by low-temperature annealing at 700 °C. For discharge rate 0.2 C, the reversible capacities 109 and 112 mAh g^−1^ were obtained for LiMn_1.95_Co_0.025_Ni_0.025_O_4_ and Li_1.1_Mn_1.95_Co_0.025_Ni_0.025_O_4_, respectively. A good cycle performance and capacity retention about 90% after 30 cycles at discharge rate 0.2–4 C were observed for the materials cycled from 3 to 4.6 V vs. Li/Li^+^. Under the same conditions pure LiMn_2_O_4_ cathode materials represent a reversible capacity 94 mAh g^−1^ and a capacity retention about 80%. Two independent experimental techniques (cyclic voltammetry at different scan rates and electrochemical impedance spectroscopy) were used in order to investigate the diffusion kinetics of lithium. This study shows that the partial substitution of Mn in LiMn_2_O_4_ with small amounts of Ni and Co allows the cyclability and the performance of LiMn_2_O_4_-based cathode materials to be improved.

## 1. Introduction

In spite of emerging power sources, Li-ion batteries still remain the most attractive ones, since their first commercial application in 1991. The cathode materials and their synthesis procedure play a tremendous role in the electrochemical performance of Li-ion batteries [1].

LiMn_2_O_4_ spinel was firstly proposed as cathode materials by Thackeray in 1983 [2] and commercialized in 1996 [3,4]. It remains one of the promising cathode materials due to its high discharge potential (4 V vs. Li/Li^+^), high safety, low cost and toxicity compared to LiCoO_2_ and LiNiO_2_ [5]. One of the substantial drawbacks of these materials is the increase in Mn^3+^ ions content during lithium intercalation at low potentials (~3 V). Upon cycling, these ions cause a significant variation of unit cell volume due to Jahn-Teller effect which leads to break the interparticle contacts [6].

This problem can be overcome by the partial substitution of Mn by other 3d-metals (Cr, Co, Fe, Ni, Cu) [7,8,9,10] which results in the enhanced structural stability. However, it is suggested that the single ion substitution for Mn^3+^ cannot resolve all the factors which cause a capacity loss [11]. A synergistic effect was reported when using double cation or multiple cation substitution of Mn^3+^ to improve the cycling life of LiMn_2_O_4_ cathode materials [12,13].

The synthesis methods of single- or multi-cation doped LiMn_2_O_4_ can be divided into conventional solid-state route [14,15,16] and *chimie douce* methods [17,18,19,20,21,22,23,24,25]. The solid-state synthesis starts with the powders of lithium hydroxide or lithium carbonate, manganese dioxide and the metal oxide dopants which are mixed and/or milled at room temperature. After that, as-prepared powder mixture is annealed at the temperature when the spinel formation is occurred. Despite the simplicity and the low-cost, this method requires rather high annealing temperatures, usually above 800 °C, in order to promote the diffusion in the solid state and to obtain a single-phase spinel. Moreover, annealing at high temperature leads to the agglomeration of particles and the formation of oxygen vacancies in the spinel lattice [26]. These features lead to the decrease in specific capacity, of cyclability and other electrochemical characteristics of doped LiMn_2_O_4_ materials.

Wet-chemical preparation techniques including sol-gel [20,21] or coprecipitation processes [19,24] allow to reduce the annealing temperature and to overcome the problems of particles agglomeration and oxygen vacancy formation due to the higher chemical homogenization of precursors. Hwang et al. synthesized the LiCo_0.1_Ni_0.1_Mn_1.8_O_4_ materials by sol–gel method and found that the phase transitions were significantly suppressed during charging and discharging. This allows the cathode materials with discharge capacity 118 mAh g^−1^ and capacity fade rate less than 10% after 40 cycles to be obtained, while the undoped LiMn_2_O_4_ phase demonstrates the capacity decrease of about 44% under the same conditions (0.3 C discharge rate) [27]. Rajakumar et al. synthesized LiCo_0.25_Ni_0.25_Mn_1.5_O_4_ spinel materials using three different chelating agents and tested their charge–discharge properties in the 3–5 V range. The utilization of oxalic acid as a chelating agent yields a LiCo_0.25_Ni_0.25_Mn_1.5_O_4_ spinel with discharge capacity of 110 mAh g^−1^ and capacity fading lower than 3% during 15 cycles [28]. Simultaneous addition of excess lithium into doped LiMn_2_O_4_ spinel allows to decrease the quantity of *d*-metal dopant, which is not active during cycling at potential range near 4 V and to decrease the Mn^3+^ concentration for the retention of high discharge capacity. Li_1.1_Mn_2−2x_Co_x_Ni_x_O_4_ (x = 0, 0.075) spinel powders were successfully synthesized using a liquid stirred tank reactor method by Liu et. al. 119 mAh g^−1^ reversible capacity and capacity retention about 94% after 80 cycles were obtained for Li_1.1_Mn_1.85_Co_0.075_Ni_0.075_O_4_ and potential range from 3 to 4.3 V [29]. Fang et al. prepared LiMn_1.95_Ni_0.025_Co_0.025_O_4_ by sol-gel mediated solid-state route at 650 °C, using highly dispersed ultra-fine Mn_3_O_4_ particles as a Mn source [30]. As-obtained cathode materials show a perfect capacity retention of about 93% at a 1 C discharge rate.

Another well-known technique which allows to obtain highly homogeneous precursor mixtures is the cryochemical (freeze drying) synthesis. A simple combination of solution freezing with the following sublimation allows to retain a uniform salt distribution obtained in the starting solution and to prepare precursors mixed at the atomic level for the synthesis of complex oxide powders. This method has been successfully used for the complex oxide preparation (high-temperature superconductors, magnetic materials, electrode materials) for a long time [31,32]. Moreover, the freeze-drying method has been extensively utilized for the preparation of LiFePO_4_ [33], LiMO_2_ (M = Mn, Ni, Co) [34,35,36,37] and other cathode materials. In the present study, the freeze drying technique was utilized for the preparation of Li_1+y_Mn_1.95_Ni_0.025_Co_0.025_O_4_ (y = 0, 0.1) cathode materials. Extra lithium was added to the material for overcoming a well-known capacity loss during the first charge of lithium-ion battery [38]. The morphology, structure and electrochemical performance of obtained samples were studied in detail. Two independent techniques (cyclic voltammetry at different scan rates and electrochemical impedance spectroscopy) were utilized for the investigation of lithium diffusion kinetics of during charge-discharge processes. The obtained results showed that the freeze-drying technique was a promising way of preparing LiMn_2_O_4_-based cathode material and a partial substitution of Mn with Co and Ni allows to improve the electrochemical performance of cathode materials due to the improvement of lithium diffusion.

## 2. Materials and Methods

### 2.1. Materials Preparation

The aqueous solutions of the following salts were used for the preparation of freeze dried precursors: Ni(CH_3_COO)_2_·4H_2_O, Co(CH_3_COO)_2_·4H_2_O, Mn(CH_3_COO)_2_·4H_2_O (Reachim, analytical grade), lithium acetate solution was prepared by dissolving Li_2_CO_3_ (Reachim, analytical grade) in the acetic acid.

Two solutions were prepared containing Li, Mn, Ni and Co in molar ratio 1.00:1.95:0.025:0.025; 1.10:1.95:0.025:0.025, respectively. The quantitative composition of all the solutions was confirmed by ICP/MS analysis (Perkin Elmer, Waltham, MA, USA). For the preparation of pristine LiMn_2_O_4_ sample, the solution containing Li and Mn acetates was prepared (Li/Mn = 1.00:2.00).

Then the solutions were sprayed into liquid N_2_ via pneumatic nozzle under continuous stirring. As-obtained cryogranules were placed in a freeze dryer chamber (Labconco Freezone 7948030 (Kansas, MO, USA)) and subjected to freeze drying at a pressure of 0.2–0.5 mbar for 72 h. Upon freeze-drying, the temperature in chamber was gradually increased from −40 to +30 °C for efficient ice removal.

The thermal treatment of freeze dried (FD) precursors was performed in air in a Nabertherm tube furnace at a heating rate of 5 °C min^−1^, then dwelling at required temperature for 4 h. The mass of the sample per run of treatment was equal to 2 g.

### 2.2. Materials Characterization

X-ray diffraction patterns for the materials obtained were registered using Rigaku D/MAX 2500 diffractometer (Tokyo, Japan) in the reflection mode (Bragg-Brentano geometry) with CuKα radiation and graphite monochromator in the 2*θ* range from 10 to 90° (scan step 0.02°; acquisition time 3 s per step). XRD data analysis and processing were performed in WinXPow software. Rietveld refinement technique was used for the determination of cell volume and cell parameter.

The morphology study was performed by scanning electron microscopy (Leo Supra 50VP, Carl Zeiss, Oberkochen, Germany) at an accelerating voltage of 21 kV and magnification ranging from ×1000 to ×100,000. The particle size distribution was determined by statistical analyses of several SEM images using the software ImageJ with a treatment of more than 200 nanoparticles.

The thermal analysis accompanied by mass-spectrometry (MS) analysis of evolved gases was performed by STA 209 PC Luxx thermal analyzer equipped with QMS 403C Aëolos mass spectrometer (Netzsch, Selb, Germany) in air by heating to 800 °C at a 10 °C min^−1^ heating rate.

The chemical composition of the obtained cathode materials was determined by ICP mass spectrometry (Perkin Elmer Elan DRC II, Waltham, MA, USA). Before analysis, the samples were dissolved in *aqua regia*. Multi element standards Perkin Elmer N9300234 and N0691579 were used for the mass spectrometer calibration.

### 2.3. Electrochemical Measurments

The cathode mass was prepared by mixing 85 wt. % of Li_1+y_Mn_1.95_Ni_0.025_Co_0.025_O_4_ with 10 wt. % of acetylene black, followed by adding 5 wt. % of polyvinylidene fluoride (Aldrich, St. Louis, MO, USA). After that, the cathode mass was placed on the stainless-steel mesh (0.05 mm thick), pressed and dried in vacuum (90 °C, ~1 mbar, 8 h). The cathode mass load was varied from 9 to 12 mg cm^−2^ and the geometric area of the cathode was equal to 1 cm^2^.

The electrochemical measurements were carried out in the three-electrode Teflon cells containing an active electrode, Li counter and Li reference electrodes separated by a porous polypropylene membrane soaked with electrolyte (1M LiPF_6_ in solution of dimethyl carbonate, diethyl carbonate, and ethylene carbonate (1:1:1 by volume)). According to the Fischer titration data, water content in the electrolyte did not exceed 25 ppm.

Galvanostatic curves were registered using the multichannel setup manufactured by the OJSC “Buster” (Sankt-Petersburg, Russia) in the potential range 3–4.6 V vs. Li/Li^+^ at room temperature. The current density during cycling was varied in the range from 20 to 400 mA g^−1^. Cyclic voltammograms (CV) were registered using Biologic VMP-3 (Seyssinet-Pariset, France) potentiostat in the potential range 3–4.7 V, the potential scan rate was varied from 100 to 2000 µV s^−1^. Electrochemical impedance spectroscopy (EIS) measurements were carried out using Solartron 1255B (Bognor Regis, UK), the frequency range was varied from 10 MHz to 1 MHz and the AC signal amplitude was 5 mV. The obtained hodographs were treated using ZView-impedance software.

## 3. Results and Discussion

### 3.1. Materials Characterization

**Figure 1:**
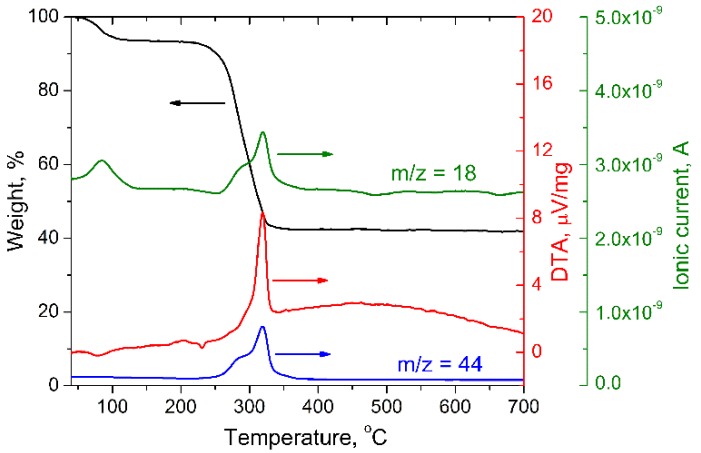
Thermal analysis results and MS-signal for mass number 44 (CO_2_) and 18 (H_2_O) for freeze dried acetate mixture.

TG (Thermogravimetry) curve of FD precursor obtained from the solution containing Li, Mn, Ni and Co acetates in molar ratio 1.1:1.95:0.025:0.025 is shown in Figure 1. It can be divided into 2 stages: a loss of absorbed and hydrated water occurs from RT (room temperature) up to 200 °C, then at 250–350 °C the acetate decomposition takes place accompanied by CO_2_ and H_2_O evolution (confirmed by MS), and well-pronounced exothermic effect. The porous structure of FD precursor facilitates both the oxygen diffusion which is necessary to transform Co(II) to Co(III) and the gas removal from the reaction zone; hence, mass loss almost finishes at 350 °C.

**Figure 2:**
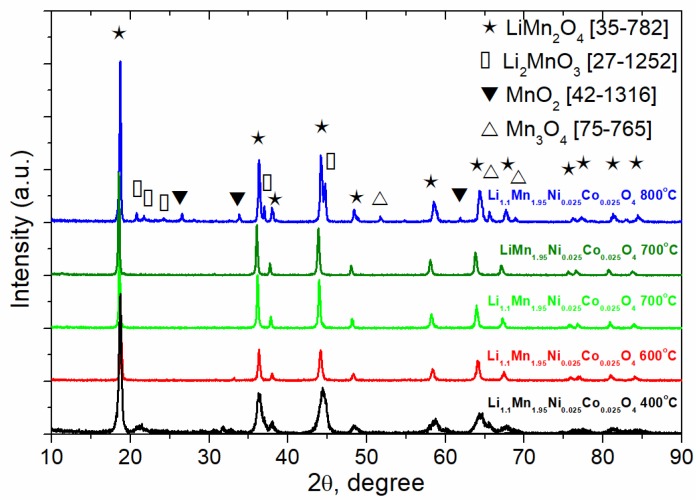
XRD patterns of Li_1.1_Mn_1.95_Co_0.025_Ni_0.025_O_4_ samples obtained at different annealing temperature and XRD pattern LiMn_1.95_Co_0.025_Ni_0.025_O_4_ sample obtained at 700 °C.

XRD analysis revealed that the target phase, substituted LiMn_2_O_4_ spinel, is formed just at 400 °C (Figure 2). However, its reflections were significantly broadened pointing at the crystallographic disordering and the small particle size, small amounts of impurities (Li_2_MnO_3_ and MnO_2_) were also detected. Hence, the heat treatment at higher temperatures was necessary to achieve the crystallographic ordering and the formation of single-phase Li_1+y_Mn_1.95_Ni_0.025_Co_0.025_O_4_. The increase in the annealing temperature to 600 °C and 700 °C resulted in narrowing the reflections in XRD pattern (Figure 2) caused by the crystallographic ordering. Further increase in the annealing temperature up to 800 °C led to the beginning of spinel decomposition indicated by the uprising reflections corresponding to Li_2_MnO_3_, MnO_2_ and Mn_3_O_4_. Based on the thermal analysis and XRD analysis results, the optimal conditions for the preparation of Li_1+y_Mn_1.95_Co_0.025_Ni_0.025_O_4_ were determined. The synthesis of Li_1+y_Mn_1.95_Co_0.025_Ni_0.025_O_4_ for futher investigation was carried out by the thermal decomposition of freeze dried precursors at 700 °C for 4 h in air (heating rate 5 °C min^−1^) and subsequent natural cooling with the furnace.

More detailed analysis of XRD patterns for the samples obtained by annealing at 700 °C showed that the main product in the both cases was Li_1+y_Mn_1.95_Ni_0.025_Co_0.025_O_4_ (y = 0; 0.1) spinel with $Fd\bar{3}m$ space group (JCPDS 35-0782). The absence of additional reflections in the diffraction pattern indicated that the lithium cations occupy tetrahedral *8a* positions while transition metal cations occupy octahedral *16d* positions. Doping of LiM_2_O_4_ spinel with Ni and Co cations led to decrease in the cell parameter *a* and in the cell volume *V* (Table 1), which could be explained by the doping of spinel with Co^3+^ and Ni^2+^ cations with average ionic radius smaller than that one of Mn^3+^ in the octahedral position and by the increase in Mn^4+^ content. The same tendency was early observed in Ref. [39]. Moreover, the introduction of extra lithium resulted in decreasing the lattice parameters, which can be explained by the allocation of lithium ions in the vacant *16c* octahedral positions while the ionic radius of lithium is smaller than that of Mn [40].

The chemical composition of the obtained samples was determined by ICP MS. The component ratio calculated from the MS analysis was equal to Li:Mn:Co:Ni = 0.98:1.94:0.025:0.024 for the initial composition LiMn_1.95_Co_0.025_Ni_0.025_O_4_ and Li:Mn:Co:Ni = 1.09:1.94:0.025:0.024 for the initial composition Li_1.1_Mn_1.95_Co_0.025_Ni_0.025_O_4_. Based on the ICP MS results, one could conclude that the molar ratio of the transition metal cations remained unchanged during the thermal treatment. A small lack of lithium could be explained by its evaporation during thermal treatment.

**Figure 3:**
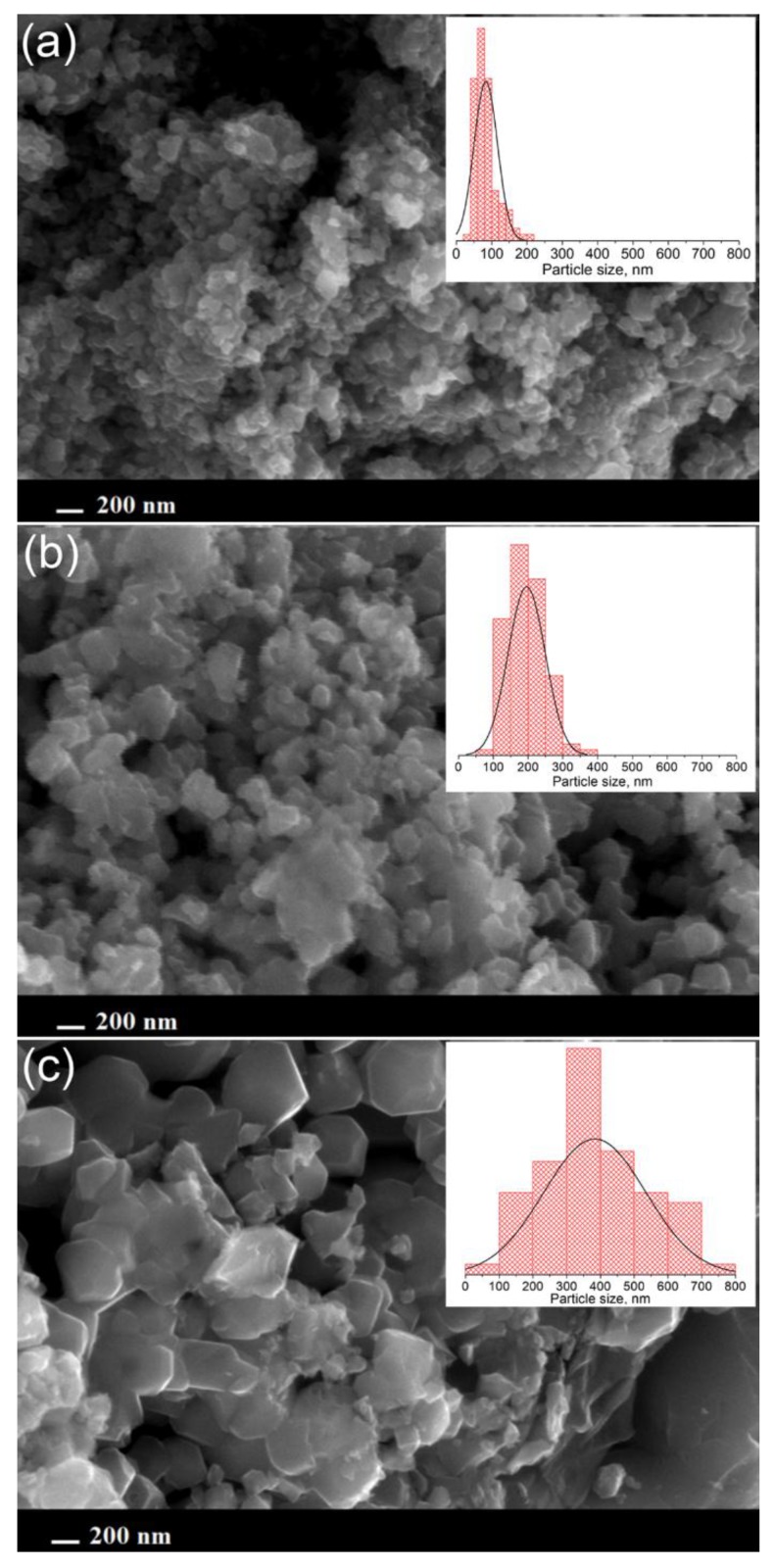
SEM micrographs of Li_1.1_Mn_1.95_Ni_0.025_Co_0.025_O_4_ obtained by annealing at 600 °C (**a**), 700 °C (**b**) and 800 °C (**c**). The particle size distributions are shown on the insets.

SEM micrographs of Li_1.1_Mn_1__.95_Co_0.025_Ni_0.025_O_4_ obtained at different temperatures (Figure 3) demonstrate that the increase in the annealing temperature leads to the enhancement of the average particle size. Annealing at 600 °С causes the formation of small plate-like particles with an average size 60–80 nm. Increasing the annealing temperature to 700 °С results in particle growing to 150–250 nm; at 800 °C, a huge increase average particle size to 500 nm occurs. It should be noted that a lithium excess did not affect significantly the particle size.

According to Scherrer equation:

(1) $$d=\frac{0.9\lambda }{\beta \cos\theta }$$

where *λ* is the CuKα wavelength (1.54 Å), *β* is the peak width at a half maximum intensity, *θ* is Bragg angle, the coherent size domain for the materials obtained at 700 °C was estimated taking account of instrumental errors, it was equal to 160 nm which coincided well with the SEM results.

### 3.2. Electrochemical Properties

**Figure 4:**
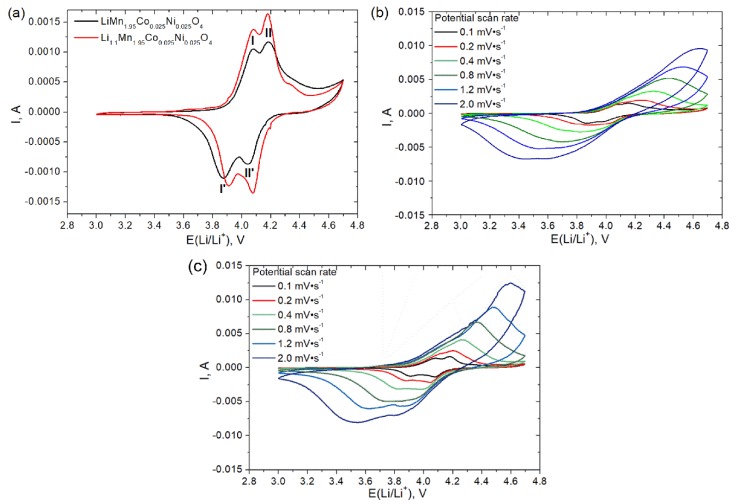
Cyclic voltammograms (CV) curve for LiMn_1.95_Co_0.025_Ni_0.025_O_4_ and Li_1.1_Mn_1.95_Co_0.025_Ni_0.025_O_4_ at the first cycle potential scan rate 0.1 mV s^−1^ (mass load was 10 mg cm^−2^ for both samples) (**a**) and CV curves for LiMn_1.95_Co_0.025_Ni_0.025_O_4_ (**b**) and Li_1.1_Mn_1.95_Co_0.025_Ni_0.025_O_4_ (**c**) obtained at different potential scan rates from 0.1 to 2.0 mV s^−1^.

The electrochemical properties of LiMn_1.95_Co_0.025_Ni_0.025_O_4_ and Li_1.1_Mn_1.95_Co_0.025_Ni_0.025_O_4_ were studied by cyclic voltammetry at a potential scan rate of 0.1 mV s^−1^ (Figure 4a). CV curves exhibited two anodic and two cathodic peaks both for LiMn_1.95_Co_0.025_Ni_0.025_O_4_ and for Li_1.1_Mn_1.95_Co_0.025_Ni_0.025_O_4_, corresponding to the deintercalation and intercalation of Li, respectively. According to [41,42], the deintercalation of lithium occurred via the following reactions:

LiMn_2_O_4_ → Li_0.5_Mn_2_O_4_ + 0.5Li ^+^(peak I)

Li_0.5_Mn_2_O_4_ → λ-MnO_2_ + 0.5Li^+^ (peak II)

And for lithium intercalation:

λ-MnO_2_+ 0.5Li^+^ → Li_0.5_Mn_2_O_4_ (peak II′)

Li_0.5_Mn_2_O_4_ + 0.5Li^+^ → LiMn_2_O_4_ (peak I′)

**Figure 5:**
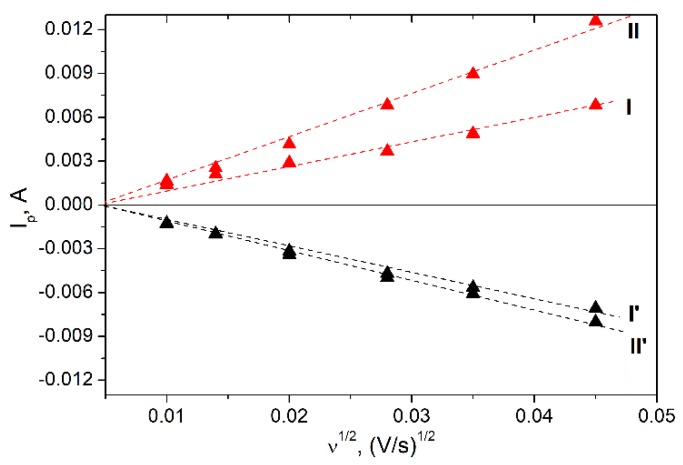
The dependence of current in CV maxima vs. square root of potential scan rate for Li_1.1_Mn_1.95_Co_0.025_Ni_0.025_O_4_ (I, II corresponds to anodic peaks at 4.07 V and 4.18 V; I’ and II’ corresponds to cathodic peaks at 3.92 V and 4.07 V, respectively).

Cyclic voltammograms for LiMn_1.95_Co_0.025_Ni_0.025_O_4_ and Li_1.1_Mn_1.95_Co_0.025_Ni_0.025_O_4_ obtained at different scan rates are shown in Figure 4b,c, respectively. In the case of low scan rate, there were two pairs of strongly pronounced peaks corresponding to the two-stage lithium intercalation/deintercalation process. Increasing the scan rate led to enhancing the electrode polarization, which result in the growing difference between intercalation and deintercalation peaks and in increasing their amplitudes. The variations of the intercalation and deintercalation peak currents vs. square root of the scan rate (Figure 5) for both cathodic and anodic branches of CV curve were linear and passed through the origin. This fact indicated the diffusion-controlled regime of lithium intercalation/deintercalation process and allowed the effective diffusion coefficients of lithium ions in the solid state to be calculated. Randles–Ševćik equation was used for the determination of Li diffusion coefficients:

(2) $$I_{p}=0.4463\cdot n^{3/2}\cdot S\cdot F\cdot {\left(\frac{F}{RT}\right)}^{1/2}\nu^{1/2}\cdot D_{ox}^{1/2}\cdot C_{ox}^{*}$$

where *n* is number of electrons, *v*—potential scan rate, V s^−1^, *F*—Faraday constant, 96500 C·mol^−1^, *R*—gas constant, 8.314 J·molK^−1^, *Т*—absolute temperature, *K*, *S*—electrode area, сm^2^, C^*^_ox_—lithium concentration in cathode material, 2.55 × 10^−2^ mol·сm^−3^. The electrode area was calculated from the weight of sample, taking into account the density and the average particle size determined from SEM image. The calculated diffusion coefficients for two intercalation and two deintercalation peaks for LiMn_1.95_Co_0.025_Ni_0.025_O_4_ and Li_1.1_Mn_1.95_Co_0.025_Ni_0.025_O_4_ are listed in Table 2.

**Figure 6:**
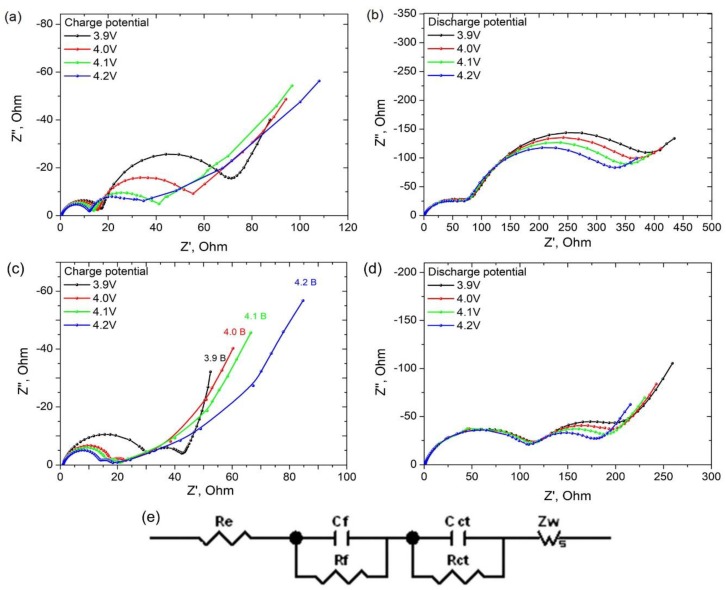
Nyquist plots for LiMn_1.95_Co_0.025_Ni_0.025_O_4_ at charge (**a**) and discharge (**b**) potentials 3.9; 4.0; 4.1 and 4.2 V and plots for Li_1.1_Mn_1.95_Co_0.025_Ni_0.025_O_4_ at the same charge (**c**) and discharge (**d**) potentials. The equivalent circuit used for the EI spectra fitting (**e**).

The lithium diffusion processes during intercalation and deintercalation were also studied by the electrochemical impedance spectroscopy. The Nyquist plots of all the samples consisted of a linear part in low frequency region, of the first semicircle at medium to high frequency region and of the second semicircle in high frequency region (Figure 6). The first semicircle in the high frequency region corresponds to the lithium migration resistance (R_f_) and capacitance of surface layer (C_f_). The second semicircle in the medium frequency region corresponds to the lithium charge transfer resistance (R_ct_) and the double layer capacitance (C_ct_). Linear part in the low frequency region corresponded to the diffusion controlled Warburg impedance W. The Nyquist plots obtained for LiMn_1__.95_Co_0.025_Ni_0.025_O_4_ and for Li_1.1_Mn_1__.95_Co_0.025_Ni_0.025_O_4_ at different potentials during charge and discharge are shown in Figure 6. The impedance spectra were fitted using the equivalent circuit model represented in Figure 6e earlier suggested in [43].

The Warburg impedance regions in Nyquist plots were used for the calculation of apparent diffusion coefficients of lithium ions:

(3) $$D={\left(\frac{V_{m}}{\sqrt{2}nFs\sigma }\;\frac{dE}{dx}\right)}^{2}$$

where *σ* is Warburg coefficient, Ohm s^−0.5^, *F*—Faraday constant, *s*—surface area, cm^2^, *V_m_*—specific molar volume, cm^3^·mol^−1^, *dE*/*dx*—potential derivative of the amount of intercalated/deintercalated lithium. The calculated diffusion coefficients for charge and discharge potential 3.9–4.2 V are listed in Table 3.

According to the literature data, the apparent lithium diffusion coefficient for LiMn_2_O_4_-based cathode material, where Mn is partly substituted by Ni [44] is varied in the range from 10^−12^ to 10^−13^ cm^2^ s^−1^ and from 10^−13^ to 10^−16^ cm^2^·s^−1^ for the materials, where Mn is substituted by Ni and Cu, respectively [45]. In our case, the lowest *D* values are equal to 2.28 × 10^−14^ and 2.60 × 10^−14^ cm^2^·s^−1^ for the potentials 3.9 and 4.1 V, respectively. So, we can conclude that the simultaneous substitution of Mn with Ni and Co using freeze-drying acetate precursors followed by low temperature heat-treatment allows to enhance the Li^+^ diffusion in comparison with substitution with only by one element (Co, Ni or Cu) using a classical solid-state preparation technique. The enhancement of lithium diffusion coefficient in materials obtained by freeze-drying can be also explained by reducing particles size in comparison with the particles obtained via solid state or sol-gel technique. The values of lithium diffusion coefficients calculated from the electrochemical impedance spectroscopy data are in good agreement with the ones calculated from cyclic voltammetry.

**Figure 7:**
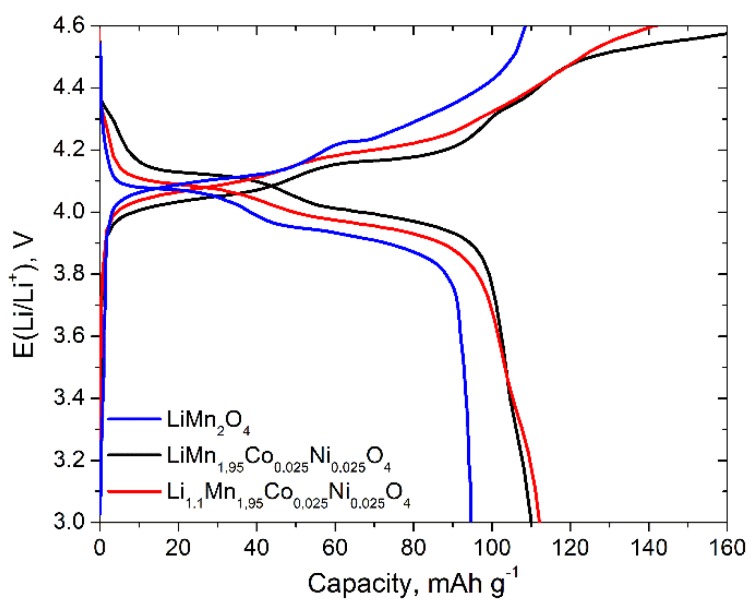
Galvanostatic charge-discharge curves for LiMn_1.95_Co_0.025_Ni_0.025_O_4_, Li_1.1_Mn_1.95_Co_0.025_Ni_0.025_O_4_ and LiMn_2_O_4_ at С/5 discharge rate at the first cycle. Potential range during cycling is 3–4.6 V.

Charge-discharge curves for LiMn_1.95_Co_0.025_Ni_0.025_O_4_, Li_1.1_Mn_1.95_Co_0.025_Ni_0.025_O_4_ and LiMn_2_O_4_ at the first cycle measured at С/5 charge-discharge rate are represented in Figure 7. The shape of galvanostatic curves also confirmed the two-stage mechanism of the lithium intercalation/deintercalation process. These two plateaus are typical for LiMn_2_O_4_ spinels. The highest discharge capacity obtained for the sample Li_1.1_Mn_1.95_Co_0.025_Ni_0.025_O_4_ at C/5 discharge rate was 112 mAh·g^−1^. This value is in good accordance with the one obtained from cyclic voltammetry at a similar rate capability. The Coulombic efficiency measured at the first cycle for all the samples is about 90%, excess charge could be attributed to the decomposition of electrolyte and formation of SEI.

**Figure 8:**
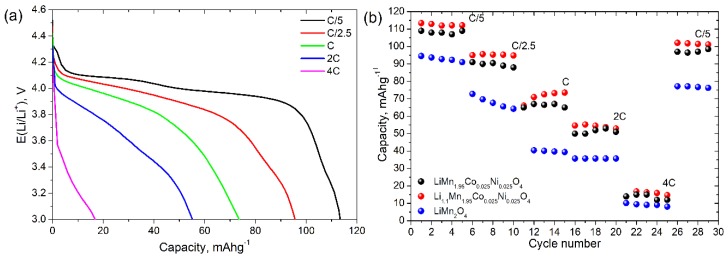
Galvanostatic discharge curves for Li_1.1_Mn_1.95_Co_0.025_Ni_0.025_O_4_ at different discharge rates (**a**) and discharge capacity of pristine LiMn_2_O_4_, LiMn_1.95_Co_0.025_Ni_0.025_O_4_ and Li_1.1_Mn_1.95_Co_0.025_Ni_0.025_O_4_ (**b**). Potential range during cycling is 3–4.6 V.

Galvanostatic curves at different discharge rates for Li_1.1_Mn_1.95_Co_0.025_Ni_0.025_O_4_ are represented in Figure 8a. The highest discharge capacity 113 mAh·g^−1^ was obtained for C/5 discharge rate. The increase in discharge current density led to the fal of the discharge capacity to 95, 75, 55 and 17 mAh·g^−1^ for discharge rates C/2.5, C, 2 C and 4 C, respectively.

Figure 8b illustrates the rate capability of LiMn_1.95_Co_0.025_Ni_0.025_O_4_, Li_1.1_Mn_1.95_Co_0.025_Ni_0.025_O_4_ and LiMn_2_O_4_ during galvanostatic cycling at different discharge rates. During the first 5 cycles at C/5, the discharge capacity of Li_1.1_Mn_1.95_Co_0.025_Ni_0.025_O_4_ was stable and equal to 112 mAh·g^−1^. The increase in discharge current density to 4 C led to the drastic fall of the discharge capacity to 17 mAh·g^−1^. However, the following decrease in the discharge rate down to C/5 after cycling at 4 C led to the recovery of discharge capacity up to 102 mAh·g^−1^. This means that the discharge capacity loss after cycling was about 10%, while for the pristine LiMn_2_O_4_ the discharge capacity loss is about 20% under the same conditions. Moreover, the excess of lithium in doped spinel allows to increase the capacity of the final material: the capacity of LiMn_1.95_Co_0.025_Ni_0.025_O_4_ exceed the capacity of Li_1.0_Mn_1.95_Co_0.025_Ni_0.025_O_4_ for all discharge rates. The doping of spinel with excess lithium undertakes the domination of Mn^4+^ on the particles surface, which suppresses the formation of Mn^2+^ on the electrode surface and improve cyclability of cathode material [46]. Also, we compare the obtained results of capacity retention with earlier published results for nickel-doped [47] and multi-doped [29] spinel. For nickel-doped spinels, the capacity retention is about 84% for the cycling program included discharging rates 0.4 C, 0.8 C, 1 C, 2 C and 3 C with the following switching back to 0.4 C rate [47]. For the multi-dopped spinels, the discharge capacity loss is about 5% after cycling in potential range 3–4.3 V, however the expansion of cycling potential range to 3–5 V leads to the drastic fall of capacity retention down to 50% [29]. So, our results are comparable and partly exceed the ones published earlier. At the same time, earlier published results on the performance of LiMn_1.95_Co_0.025_Ni_0.025_O_4_ cathode materials prepared by sol-gel mediated solid-state route are better in comparison with ours, however the preparation procedure described in ref. [30] is quite complicated and includes more than two stages with annealing and milling of precursors. So, one can conclude that the partial substitution of Mn with Ni and Co leads to the improvement of the electrochemical stability of Li-Mn spinels.

## 4. Conclusions

LiMn_1.95_Co_0.025_Ni_0.025_O_4_ and Li_1.1_Mn_1.95_Co_0.025_Ni_0.025_O_4_ cathode materials were obtained via freeze drying of acetate precursors followed by thermal treatment. Well-formed single-phase spinel was obtained at 700 °C. For 0.2 C discharge rate, the reversible capacities 109 and 112 mAh·g^−1^ were obtained for LiMn_1.95_Co_0.025_Ni_0.025_O_4_ and Li_1.1_Mn_1.95_Co_0.025_Ni_0.025_O_4_, respectively, which is higher than the ones obtained for pure LiMn_2_O_4_ prepared under the same conditions (94 mAh·g^−1^). The diffusion kinetics of lithium during charge-discharge processes was determined via two independent techniques: cyclic voltammetry at different scan rates and electrochemical impedance spectroscopy. The obtained value of diffusion coefficients shows that the partial substitution of Mn simultaneously with Co and Ni allows to improve the electrochemical performance of cathode materials due to the improvement of lithium diffusion kinetics. A good cycle performance and capacity retention about 90% after 30 cycles at discharge rate 0.2–4 C were obtained when the cathode materials were cycled from 3 to 4.6 V. The study shows that the partial substitution of Mn with a small amount of Ni and Co in LiMn_2_O_4_ structure allows to improve the electrochemical properties and LiMn_1.95_Co_0.025_Ni_0.025_O_4_ and Li_1.1_Mn_1.95_Co_0.025_Ni_0.025_O_4_ prepared by freeze-drying technique will be promising materials for lithium ion batteries.

## Figures and Tables

**Table 1:** Structural parameters of the spinel samples obtained by annealing at 700 °С.

Sample	*a*, Å	*V*, Å^3^
LiMn_2_O_4_	8.2390 (5)	559.27 (5)
LiMn_1.95_Co_0.025_Ni_0.025_O_4_	8.2301 (5)	557.46 (5)
Li_1.1_Mn_1.95_Co_0.025_Ni_0.025_O_4_	8.2200 (5)	555.42 (6)

**Table 2:** The calculated value of lithium ion diffusion coefficient in LiMn_1.95_Co_0.025_Ni_0.025_O_4_ and Li_1.1_Mn_1.95_Co_0.025_Ni_0.025_O_4_.

Sample	Potential, *V*	Peak I’	Peak II’	Peak I	Peak II
LiMn_1.95_Co_0.025_Ni_0.025_O_4_	E (Li/Li^+^), V	3.88	3.98	4.07	4.18
	D_Li+,_ cm^2^ s^−1^	1.00 × 10^−14^	3.27 × 10^−14^	1.34 × 10^−14^	1.47 × 10^−14^
Li_1.1_Mn_1.95_Co_0.025_Ni_0.025_O_4_	E (Li/Li^+^), V	3.92	4.07	4.07	4.18
	D_Li+,_ cm^2^ s^−1^	1.01 × 10^−14^	2.39 × 10^−14^	1.27 × 10^−14^	1.92 × 10^−14^

**Table 3:** Li^+^ apparent diffusion coefficients at different charge and discharge potentials in LiMn_1.95_Co_0.025_Ni_0.025_O_4_ and Li_1.1_Mn_1.95_Co_0.025_Ni_0.025_O_4_.

Sample	Potential, *V*	3.9	4.0	4.1	4.2
LiMn_1.95_Co_0.025_Ni_0.025_O_4_	Charge, D_Li+,_ cm^2^ s^−1^	4.49 × 10^−12^	1.09 × 10^−12^	4.11 × 10^−13^	2.84 × 10^−13^
	Discharge, D_Li+,_ cm^2^ s^−1^	2.28 × 10^−14^	2.73 × 10^−14^	2.60 × 10^−12^	2.90 × 10^−12^
Li_1.1_Mn_1.95_Co_0.025_Ni_0.025_O_4_	Charge, D_Li+,_ cm^2^ s^−1^	1.42 × 10^−12^	1.19 × 10^−12^	9.09 × 10^−13^	4.10 × 10^−13^
	Discharge, D_Li+,_ cm^2^ s^−1^	5.33 × 10^−14^	7.99 × 10^−14^	6.31 × 10^−14^	7.33 × 10^−14^
